# Optically Induced Irreversible Ferroelastic and Ferroelectric Switching in Epitaxial BaTiO_3_ Films on Silicon

**DOI:** 10.1021/acsnano.5c05309

**Published:** 2025-10-23

**Authors:** Ibukun Olaniyan, Alfredo Blázquez Martínez, Valentin Väinö Hevelke, Sven Wiesner, Rong Wu, Thanh Luan Phan, Robin Cours, Nikolay Cherkashin, Sylvie Schamm-Chardon, Dong-Jik Kim, Catherine Dubourdieu

**Affiliations:** † Helmholtz-Zentrum Berlin für Materialien und Energie, Hahn-Meitner Platz 1, 14109 Berlin, Germany; ‡ Freie Universität Berlin, Physical and Theoretical Chemistry, Arnimallee 22, 14195 Berlin, Germany; § CEMES-CNRS and Université de Toulouse, 29 Rue Jeanne Marvig, F-31055 Toulouse, France

**Keywords:** barium titanate, ferroelastic switching, polarization switching, UV, photoelectric effect, silicon, Raman

## Abstract

Optical manipulation of ferroelectric polarization is a promising method for potentially ultrafast and remote polarization switching without electrodes. Here, we report optical ferroelastic and ferroelectric switching by UV irradiation in epitaxial BaTiO_3_ thin films grown on a SrTiO_3_-buffered Si substrate. The pristine BaTiO_3_ film is in the tetragonal ferroelectric phase with both in-plane and out-of-plane ferroelectric polarization. After irradiation by a 325 nm UV laser, the polarization is mainly out-of-plane indicating ferroelastic switching. Moreover, all initial downward polarized domains have switched to upward, thus showing ferroelectric 180°-domain switching. After irradiation the film exhibits mainly a single up-oriented polarization and as a result, the irradiated regions exhibit an enhanced piezoelectric response. We propose that the observed ferroelastic and ferroelectric switching is triggered by additional strain/stress fields generated by internal electric fields arising mainly from the spatial charge carrier separation after photoexcitation. These strain/stress fields add up to the Vegard strain field and to local heating, which induce defect motion and a final state with full strain relaxation. This optical switching enables remote manipulation of ferroelastic and ferroelectric domains in BaTiO_3_ films on silicon. Moreover, UV illumination appears as a potential postdeposition treatment to heal defects and obtain a strain-free epitaxial layer.

## Graphical abstract

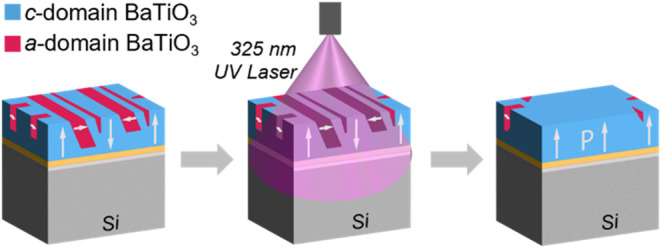

## Introduction

In ferroelectric thin films, switchable polarization is key to the functionality of devices such as nonvolatile memories, optoelectronic components, and sensors.^1–3^ Polarization switching is mainly realized by the application of an electric field, either through contacting an electrical needle to a top electrode of a device or through contacting a conductive tip to the samples (with or without a top electrode).^4^ The application of a mechanical (inhomogeneous) stress is another way to switch the polarization^5^ but is impractical for device applications. Optical switching is an attractive way to manipulate the ferroelectric polarization as it is contactless and there is no risk of electrical breakdown compared to electrical switching.

Light-ferroelectric interactions give rise to a rich variety of phenomena, including ferroelectric polarization switching,^6–11^ structural phase transitions,^12–14^ domain wall motion,^15,16^ lattice deformation,^17–21^ modification of imprint field,^22,23^ ferroelastic switching,^24–26^ paraelectric to ferroelectric phase transition,^27,28^ and the emergence of nontrivial polar textures.^29–31^ These light-induced phenomena are driven by electronic or thermal effects. Electronic effects involve the generation of photoexcited carriers, resulting in photostriction,^20,21^ (bulk) photovoltaic effects,^6,32^ screening of the depolarization field,^18,19^ and modifications of the interfacial band structure.^7,9^ Thermal effects arise from laser-induced heating, which generates internal electric fields via thermoelectric or pyroelectric mechanisms, thereby influencing ferroelectric properties.^13,14,33^ These processes can coexist and may occur simultaneously across different time scales ranging from subpicoseconds to seconds.

Ferroelectric polarization switching by optical means has been demonstrated in Pb­(Zr,Ti)­O_3_ films grown on DyScO_3_ ^7^ and LaAlO_3_.^8^ Coupled ferroelectric/ferroelastic switching has been shown in BiFeO_3_ films grown on LaAlO_3_ ^13,14^ and TbScO_3_.^6^ For BaTiO_3_, light-induced ferroelectric switching has been reported in thin films grown on SrTiO_3_ ^9,11^ and LaAlO_3_.^10^ Transient domain wall motion^15,16^ and ferroelastic switching^24–26^ were reported in BaTiO_3_ single crystals. Photostriction in BaTiO_3_ membranes^21^ and a photoinduced phase transition in BaTiO_3_ nanowires were observed.^12^

In this work, we demonstrate ferroelastic switching under UV irradiation from *a*- to *c*-domains as well as 180° ferroelectric switching in BaTiO_3_ thin films epitaxially grown on silicon. A 325 nm UV laser is used to provide the optical stimulus and to probe the crystalline structure by Raman spectroscopy. Under increasing laser beam intensity, the number of *a*- to *c*-switched domains increases. The *c*-domains are fully up-oriented as shown by piezoresponse force microscopy. Crystalline changes are evidenced by scanning transmission electron microscopy and strain analysis. UV irradiation leads to dislocations movement down to the interface with SrTiO_3_, to an improved crystallinity (lower mosaicity) and to a homogeneous strain relaxation throughout the film—except at the interface with SrTiO_3_—to the bulk *c*-axis oriented state. Above band gap excitation is required to trigger these effects. The proposed mechanisms involve the creation of strong strain/stress fields upon illumination which, together with local heating, might be the driving force for defect motion and the phase evolution toward its bulk state. Our results provide a route toward the optical control of the polarization in a Pb-free ferroelectric on silicon.

## Results

### Structural Characterization of the Nonirradiated Heterostructure

An epitaxial 20.8 nm BaTiO_3_ film was grown on a single-crystalline Si(100) substrate with a 3.6 nm SrTiO_3_ layer template by molecular beam epitaxy (Figure 1a). The growth details are given in the Method Section. The thicknesses were determined by X-ray reflectometry (Figure 1b). Note that the thin SiO_*x*_ layer is formed after the epitaxy of SrTiO_3_ on Si (thus not disrupting the epitaxial growth of BaTiO_3_) by oxygen diffusion to the Si substrate mostly during the BaTiO_3_ growth at 650 °C.^34,35^ The X-ray diffraction θ/2θ, in-plane and phi scans are shown in Figure 1b-d. The typical 45° rotated “cube on cube” epitaxial relationship for BaTiO_3_ and SrTiO_3_ relatively to Si is observed ([110]­BaTiO_3_//[110]­SrTiO_3_//[100]­Si and (001)­BaTiO_3_//(001)­SrTiO_3_//(001)­Si). The average out-of-plane and in-plane lattice parameters are of 4.067 Å and 3.976 Å respectively (for bulk BaTiO_3_, *c*_bulk_ = 4.036 Å and *a*_bulk_ = 3.994 Å).^36^ The strain pattern in the film will be discussed later.

**Figure 1:**
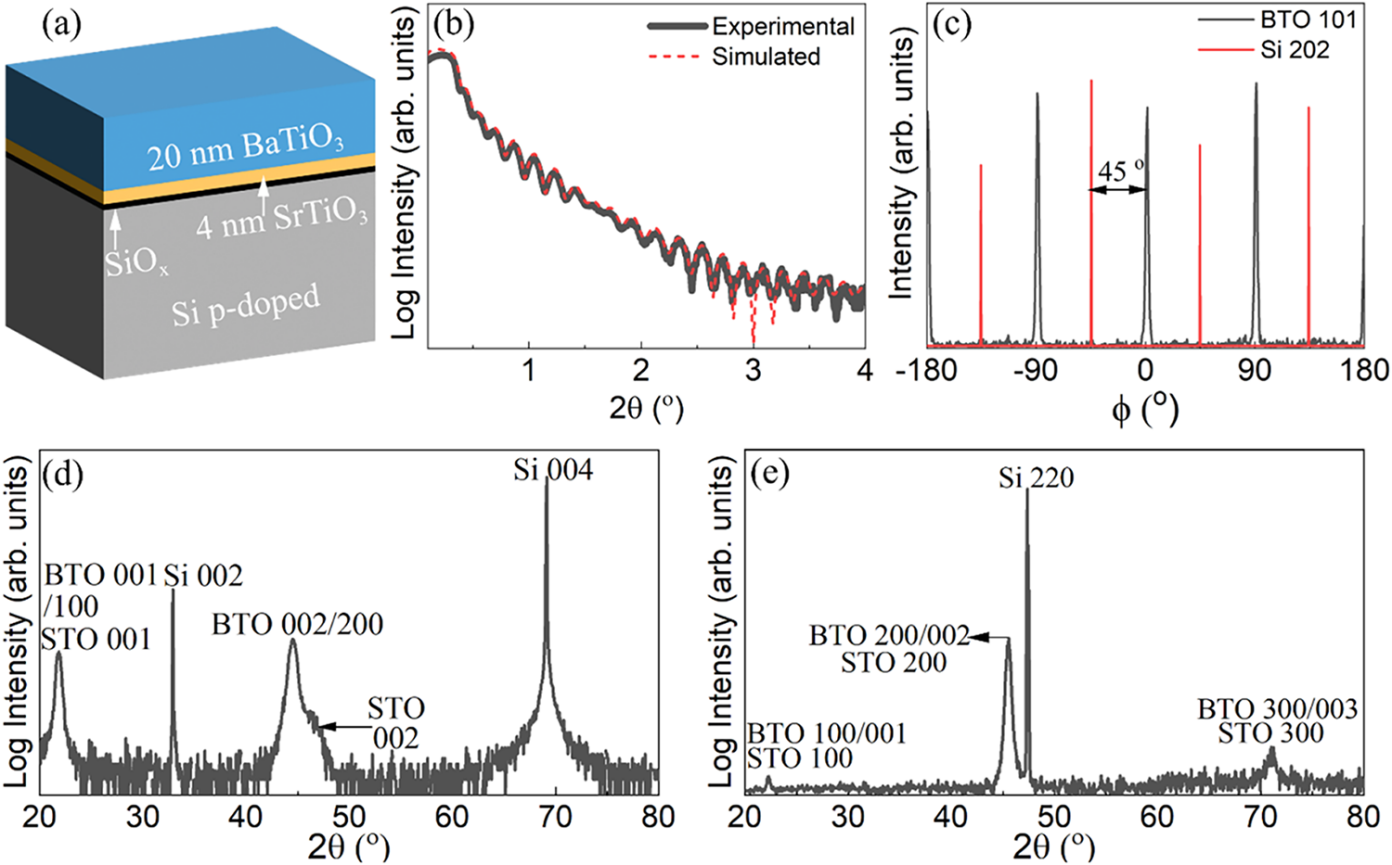
(a) Schematics of the stack with nominal thicknesses. (b) X-ray reflectivity of the sample, indicating a thickness of 20.8 nm for BaTiO_3_ and 3.6 nm for SrTiO_3_. (c) XRD Phi scan of BaTiO_3_ 101 and Si 202 reflections. (d) XRD θ–2θ pattern and (e) in-plane XRD pattern of the sample.

Raman analysis shows that the as-grown BaTiO_3_ is in the tetragonal phase and consists of mixed *a*- and *c*-domains (with the long axis of the tetragonal cell lying in-plane and out-of-plane, respectively). The Raman spectra shown in Figure 2a were measured using a UV laser without polarization optics (unpolarized configuration) and in a parallel-polarized configuration, denoted as *Z*(*XX*)*Z̅* in Porto notation. Here, *Z* corresponds to the surface normal, aligned with Si[001] direction, and *X* is aligned with BaTiO_3_[110] direction (Si[100] direction) as illustrated in Figure S1 of the Supporting Information. This polarization geometry suppresses the Si Raman mode^37^ thereby allowing a clear visualization of the BaTiO_3_ mode at 520 cm^–1^. The observed BaTiO_3_ Raman modes at 301 cm^–1^, 475 cm^–1^, 520 cm^–1^ and 725 cm^–1^, correspond to the B_1_ + E­(LO + TO), A­(LO_2_) + E­(LO_3_), A_1_(TO_3_), and A­(LO_3_) + E­(LO_4_) modes, respectively.^38^ According to the Raman selection rules, E­(LO) modes should be absent in the backscattering geometry because their polarization direction is parallel to the wave vector. However, the objective we used with a numerical aperture of 0.49 can introduce a finite collection angle, which allows the observation of otherwise forbidden modes due to deviations from ideal backscattering conditions.^39^ The B_1_ + E­(LO + TO), A­(LO_2_) + E­(LO_3_), A_1_(TO_3_) and A­(LO_3_) + E­(LO_4_) modes are characteristic of a tetragonal ferroelectric BaTiO_3_ thin film.^38,40^ Among these modes, the A­(LO_2_) + E­(LO_3_) and A­(LO_3_) + E­(LO_4_) modes are characteristic of *c*-domains, and the A_1_(TO_3_) mode is indicative of *a*-domains.^15,25^

**Figure 2:**
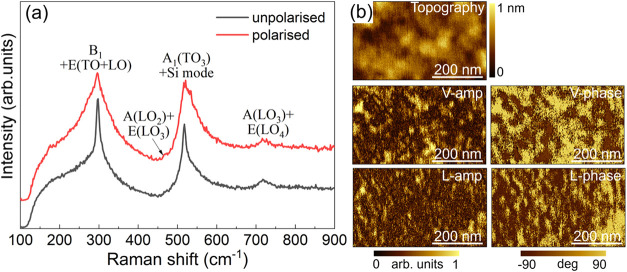
(a) Unpolarized (black spectrum) and parallel-polarized (red spectrum) Raman spectra of the BaTiO_3_/SrTiO_3_/SiO_*x*_/Si heterostructure. (b) PFM images showing topography, vertical amplitude (V-amp), vertical phase (V-phase), lateral amplitude (L-amp) and lateral phase (L-phase) of the same heterostructure.

Piezoresponse force microscopy (PFM) images show that the BaTiO_3_ layer has both vertical and lateral polarization components (Figure 2b), which is consistent with the Raman analysis. In the vertical phase image, the observed bright and dark phase contrasts correspond to downward (P_down_) and upward (P_up_) polarization directions, respectively. The up and down components are randomly distributed, which is typical for pristine MBE BaTiO_3_/SrTiO_3_ on Si.^41^ The MBE growth conditions favor a pristine state with no preferred orientation. However, once a bias is applied, a preferred-up polarization emerges (polarization pointing toward the BaTiO_3_ top surface). This is indicated by the vertical imprint of the hysteresis loops measured by switching spectroscopy PFM (SSPFM) at different locations of the surface (Figure S2a of the Supporting Information). This asymmetry in polarization may be attributed to internal built-in fields, originating from asymmetry in charge screening ability by the top (air) and bottom (SrTiO_3_/SiO_*x*_/Si) interfaces, strain gradient, defect dipoles, or trapped charges. The preferred-up polarization might also originate from a nonswitching (pinned) part of the film.

Up or down domains can be written and switched as shown in Figure S2b, where two rectangular regions were poled with opposite DC electric fields (+7 V and −7 V); the amplitude between opposite polarization is similar and the phase difference between the domains is 180°. Back-switching of *P*_down_ poled regions to *P*_up_ occurs, indicating an asymmetry in the system.

### Ferroelastic Switching from *a*-Domains to *c*-Domains under UV Irradiation

Hollow-square-shaped patterns on the BaTiO_3_ surface were created to easily locate the regions of interest (Figure S3 of the Supporting Information). UV irradiation experiments were performed on different locations of the sample at different laser powers of 0.3, 0.6, 1, 2.5, 5, and 10 mW/μm^2^ and corresponding exposure times of 500, 250, 150, 60, 30, and 15 s, respectively, such that the fluence remained constant at 150 mJ/μm^2^. Although the theoretical laser spot size is of ∼810 nm (value used for the fluence calculation), the effective irradiated regions extended up to ∼5 μm due to the Gaussian intensity distribution of the laser beam. Pronounced changes in the Raman spectra were observed after UV irradiation with increasing laser power intensity (Figures 3a and S4). These changes include the emergence of a Raman mode at 195 cm^–1^ (marked with an asterisk), a change in the Raman spectra between 200 and 270 cm^–1^ from a concave to a convex shape, an increase in the intensity of the A­(LO_2_) + E­(LO_3_) and A­(LO_3_) + E­(LO_4_) modes, and a decrease in the intensity of the A_1_(TO_3_) mode. The Raman mode at 195 cm^–1^, the convex shape of the spectra between 200 to 270 cm^–1^ (see the shaded gray rectangular area in Figure 3a) and the enhanced intensities of the A­(LO_2_) + E­(LO_3_) and A­(LO_3_) + E­(LO_4_) modes^25^ are all attributed to the increase of *c*-domains as the same features are observed in a *c*-oriented BaTiO_3_ single crystal but not in an *a*-oriented one (Figure S5). In parallel, the decrease in intensity of the A_1_(TO_3_) mode indicates a reduction in *a*-domains (Figure S5). The progressive increase of *c*-domains at the expense of the *a-*ones is illustrated in Figure 3b (the Gaussian fits of the Raman modes between 450 and 900 cm^–1^ are shown in Figure S6). These findings provide a structural proof of ferroelastic switching from *a*- to *c*-domains upon UV irradiation.

**Figure 3:**
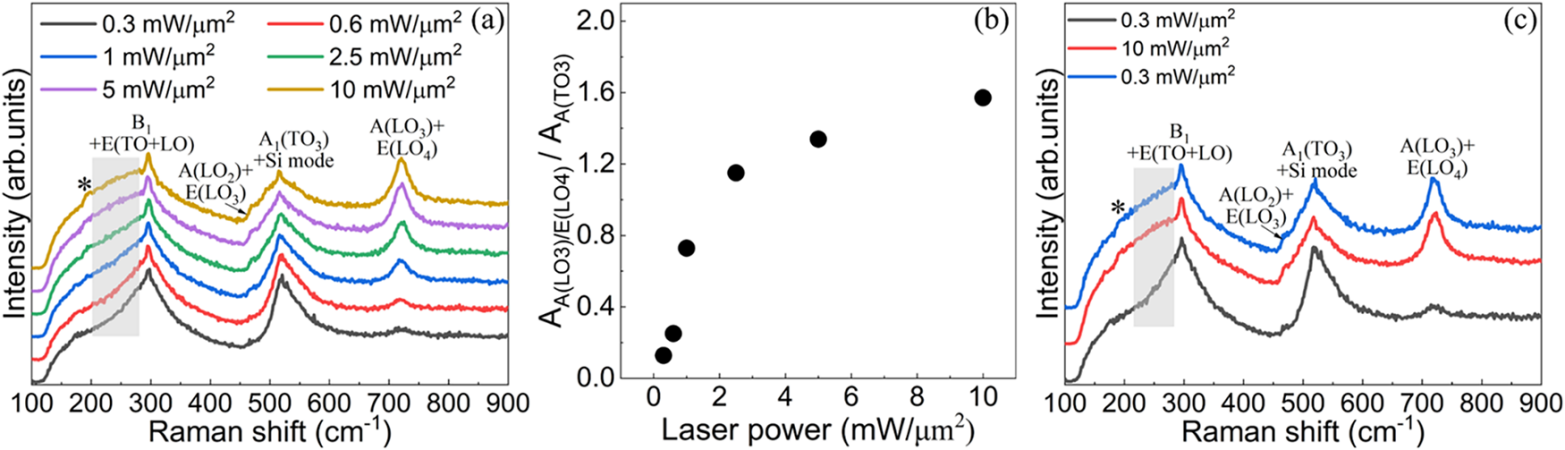
(a) Parallel-polarized Raman spectra showing the effect of the laser irradiation on BaTiO_3_ with different laser intensities (each measurement is done in a different location). All experiments are done with the same fluence of 150 mJ/μm^2^. The shaded rectangular area highlights the transition from a concave to a convex shape. (b) The intensity ratio of the A­(LO_3_) + E­(LO_4_) mode to the A­(TO_3_) mode as a function of laser intensity. (c) Parallel-polarized Raman spectra of a region measured in a sequence of 0.3 mW μm^–2^ (black spectra), 10 mW μm^–2^ (red spectra), and 0.3 mW μm^–2^ (blue spectra) laser intensity in the same location (each measurement is done with a deposited energy of 150 mJ/μm^2^).

Consecutive Raman spectra of the same region were recorded at laser intensities of 0.3, 10, and back to 0.3 mW μm^–2^ (Figure 3c), with a fluence of 150 mJ/μm^2^ for each illumination. The Raman spectra obtained in the last two measurements (i.e., 10 and 0.3 mW μm^–2^) are identical, indicating the stability of the ferroelastically switched domains. The observed ferroelastic switching persisted for a test period of 8 months. Hence, the ferroelastic switching by laser irradiation is stable and irreversible.

All experiments were carried out using laser intensities and fluences below the damage threshold. Figure S7a,b show the evolution of the Raman spectrum in regions exposed to different fluences–up to 3 J/μm^–2^–with 0.3 and 10 mW/μm^2^ laser power, respectively. The absence of spectral degradation such as peak broadening of Raman modes indicates that the damage threshold of the BaTiO_3_ layer exceeds 3 J/μm^2^.

Increasing the fluence at a constant power intensity of 0.3 mW/μm^2^ led to progressive ferroelastic switching (Figure S7a). However, when comparing regions irradiated with a similar fluence of 3 J/μm^2^ at 0.3 and 10 mW/μm^2^ (Figure S7c), a more pronounced decrease in *a*-domain and increase in *c*-domain signatures were observed in the region exposed to 10 mW/μm^2^. This observation indicates that the ferroelastic switching is governed not only by the total fluence but also by the laser power intensity.

### Ferroelectric 180° Switching of the UV Laser Irradiated Regions

PFM reveals enhanced electromechanical piezoresponse in the irradiated regions (Figure 4a), with uniform P_up_ orientation as indicated by the dark contrast in the V-phase images (Figure 4b). Hence, UV irradiation also triggers 180° ferroelectric switching toward a uniform P_up_ polarization. The switched P_down_ to P_up_ polarization component is stable for a test period of 8 months. This orientation corresponds to the preferred orientation of polarization when recording SSPFM loops (Figure S2a). The 180° ferroelectric switching toward P_up_ after UV illumination is also clearly observed in Figure S8.

**Figure 4:**
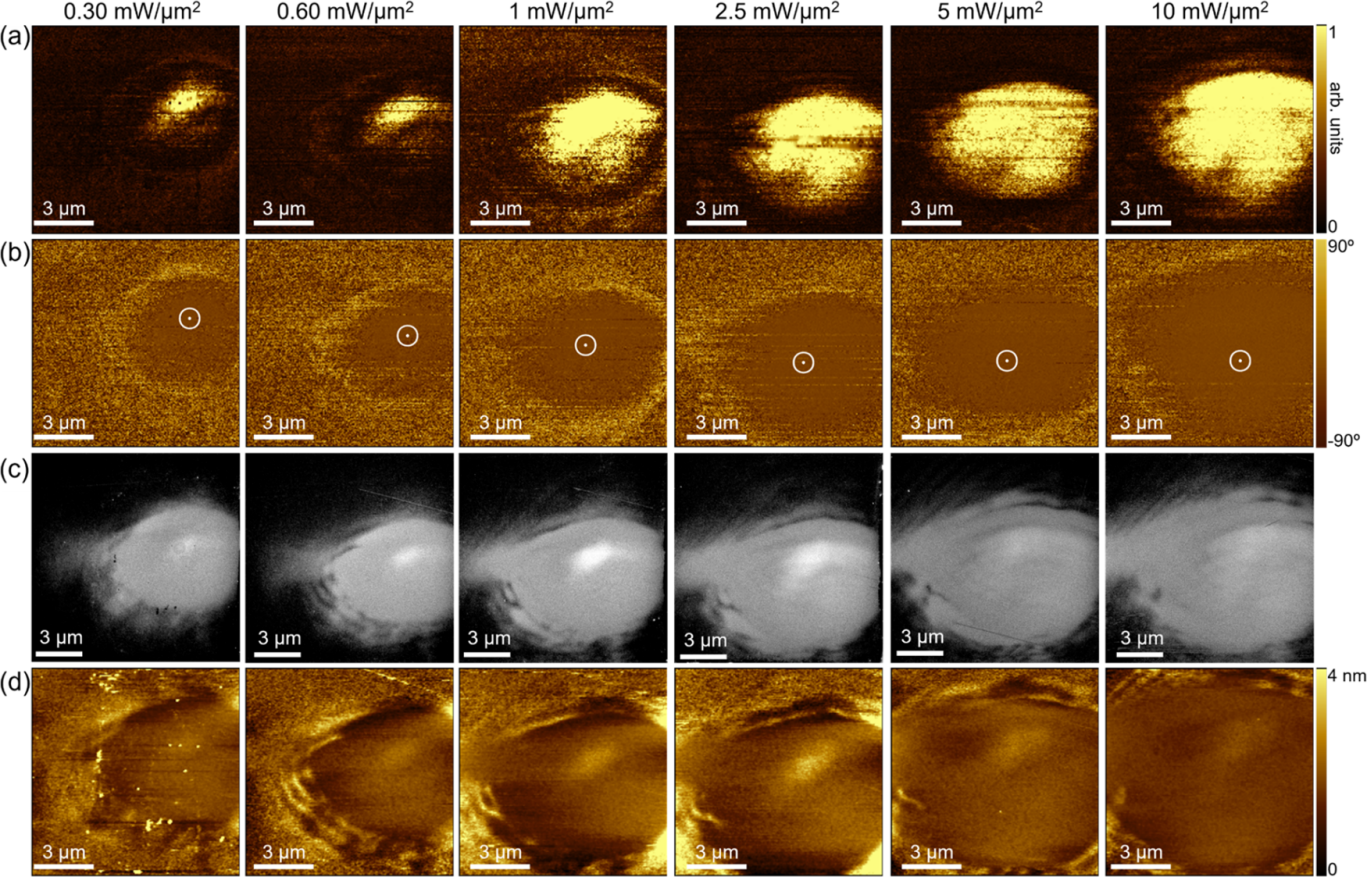
PFM images of regions irradiated with different laser powers showing (a) vertical amplitude and (b) vertical phase responses. (c) SEM images and (d) AFM images of the same irradiated regions.

Figure S9a shows a region exhibiting an increased PFM amplitude with a fully up-oriented polarization (V-phase) after exposure to the UV laser at 10 mW μm^–2^. As shown in Figure S9b with poling experiments, the polarization in the irradiated regions remains switchable but back-switching is strong when poling the polarization down, indicating a preferred-up polarization state. To exclude that the boundary conditions are responsible for the back-switching, we performed similar experiments on a larger area of 60 × 60 μm^2^ (Figure S10). Note that back-switching was already observed in the pristine film (Figure S2b). This is not clear whether it is enhanced by the UV illumination (much more statistics would be needed to conclude).

### Macroscopic and Microscopic Changes of the Surface and Crystalline Structure

We observed changes of the irradiated surfaces as seen from scanning electron microscopy (SEM) and atomic force microscopy (AFM). The irradiated regions in the SEM images show brighter contrast compared to the surrounding areas (Figure 4c), which directly correlates with the AFM images (Figure 4d). The line profiles of the AFM images reveal that the laser irradiation creates a hill-like feature with a height of ∼1.5 nm (Figure S11). As this value is rather low, the SEM contrast is unlikely to be solely due to a topographical change. Given that the SEM contrast is also associated with atomic numbers of elements and the channelling effect of electrons with respect to crystallographic lattice planes,^42^ there might be a modification of chemistry, strain, or lattice distortion in the irradiated regions.

High-angle annular dark-field (HAADF) scanning transmission electron microscopy (STEM) was used to image the Ba, Sr, and Ti atomic columns along the ⟨010⟩ direction within the nonirradiated and irradiated samples (Figure 5a,5b). AbStrain procedure was applied for correction of distortions and calibration errors in the images^43^ and for extraction of the strain tensor components with reference to the bulk *c*-oriented BaTiO_3_ cell. The reddish (bluish) color indicates a tensile (compressive) strain while the greenish color corresponds to zero strain. The relative displacement of Ti atoms with respect to the barycenter of the Ba­(Sr) cell (presented in form of arrows), which is colinear with the polarization, was extracted by Relative displacement procedure.^43^

**Figure 5:**
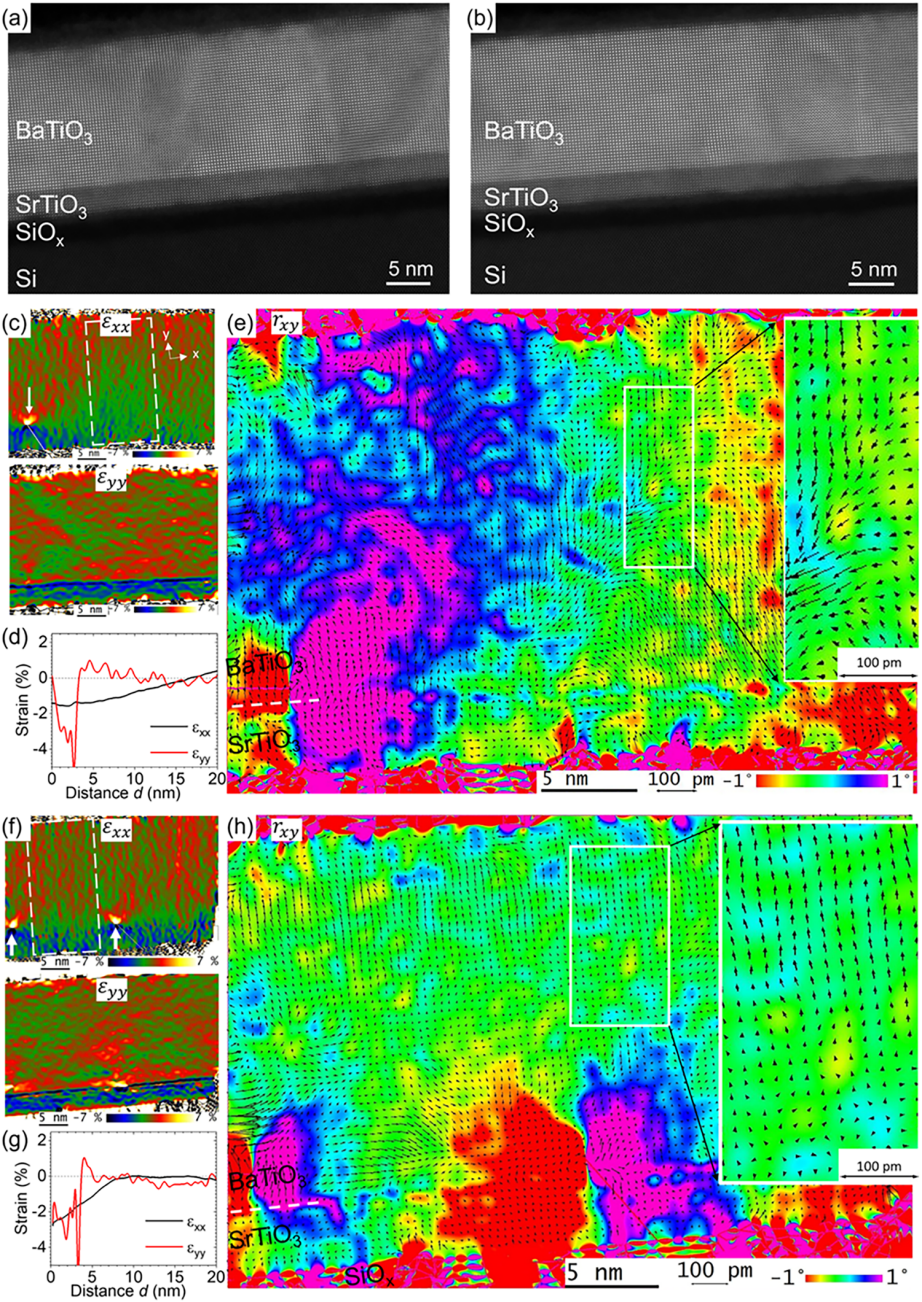
High resolution HAADF STEM image of the BaTiO_3_/SrTiO_3_/SiO_*x*_/Si heterostructure in (a) nonirradiated region, and (b) irradiated region. AbStrain and Relative displacement analysis of the HAADF-STEM images corresponding to nonirradiated (c–e), and irradiated regions (f–h) of the BaTiO_3_/SrTiO_3_ heterostructure on Si: (c, f) in-plane (ε_*xx*_) and out-of-plane (ε_*yy*_) strain tensor components with reference to the *c*-axis bulk BaTiO_3_ cell; (d, g) strain profiles extracted from the white rectangular depicted in (c, f). (e, h) Colored background: rigid body rotation map. Arrows: relative displacement of Ti atoms with respect to the barycenter of the Ba­(Sr) cell. The scale bar for the relative displacement amplitude is shown by double arrow.

Figure 5c shows the in-plane and out-of-plane strain components obtained from the nonirradiated area. The strain varies both laterally and throughout the thickness, which is typical for BaTiO_3_ on SrTiO_3_-buffered Si.^4^ The BaTiO_3_ film typically contains dislocations at the interface with SrTiO_3_ but also inside the film, particularly along tilted regions. The average size of coherent domains (separated by low angle grain boundaries corresponding to the mosaicity) is typically of ∼15 nm. Figure 5d displays the strain profiles extracted from the region marked by the white rectangular in Figure 5c. The quantitative lattice profile analysis (in between dislocations) indicates that the BaTiO_3_ layer is fully *c*-domains in the first 5–6 nm and that it evolves to mixed *c*- and *a*-domains, with a high heterogeneity laterally. Polarization domains are of a few nanometers in size laterally. Moreover, the strain and the polarization are highly disturbed in the vicinity of dislocations.

The laser irradiation has a strong impact on the strain. The strain distribution appears rather homogeneous (Figure 5f–h). The dislocations (arrows in Figure 5f) are mostly pinned right above the BaTiO_3_/SrTiO_3_ interface and not in the film anymore. They obey the in-plane ⟨100⟩ Burger’s vector. Consequently, the size of the coherent crystalline domains has doubled to about 30 nm. The greenish color of both strain components above the dislocations (Figure 5f) indicates zero strain, meaning that the film is fully relaxed (except for the first 3–4 nm above the interface) relatively to the *c*-axis bulk BaTiO_3_ unit cell. This finding is confirmed by the strain profiles (Figure 5g) extracted from the region marked by the white rectangular in Figure 5f: the BaTiO_3_ layer is fully relaxed almost through the whole thickness. The rigid body rotation map reflects the much-improved homogeneity and crystalline quality (Figure 5h versus Figure 5e). Above the perturbed region due to dislocations at the interface, the Ti displacement in BaTiO_3_ is either close to zero (presence of *a*-domain with the *c*-axis pointing in the *z* direction), or pointing up, with an amplitude of ∼20 pm, in line with the observed PFM results.

EELS measurements were performed on nonirradiated and irradiated regions, but no conclusions could be made on a possible change in stoichiometry. More statistics and a better resolution in energy would be needed.

## Discussion

A first question that is raised when illuminating a material under light is whether the beam is inducing heating of the material. A previous study suggested that laser-induced changes in the Raman spectra of a BiFeO_3_ film on a LaAlO_3_ substrate were due to thermal effects.^13^ Here, thermal effects alone can not be responsible for the observed ferroelastic/ferroelectric switching. Temperature-dependent Raman spectra from 300 to 500 K (Figure S12) show that the increase in temperature does not lead to an increase of the intensity of A­(LO_2_) + E­(LO_3_), A_1_(TO_3_), A­(LO_3_) + E­(LO_4_) and B_1_ + E­(LO + TO) modes. Moreover, thermal effects are also present under violet or red laser irradiations, as the underlying Si substrate can absorb these wavelengths and indirectly heat the BaTiO_3_ layer. Experiments carried out using violet and red lasers, with energies of 3.06 and 1.96 eV, respectively, did not lead to ferroelastic or ferroelectric switching (Figure S13). Hence, the use of a laser with energy above the bandgap of the BaTiO_3_ film is key in triggering the polarization switching (the band gap is measured at 3.36 eV by spectroscopic ellipsometry, Figure S14). Note that the increased bandgap in our film as compared to that of bulk BaTiO_3_ (∼3.2 eV)^44,45^ can be attributed to strain state of the BaTiO_3_ film.^44^ A bandgap of 3.35 eV has been reported in a 20 nm thick BaTiO_3_ film deposited on SrTiO_3_ substrate.^44^ Mechanisms at play must therefore follow-up the generation of electron–hole pairs (electrons moving from the valence band to the conduction band of BaTiO_3_).

In contrast to most reports on light-induced effect in ferroelectrics, we provide also local structural/strain analyses before and after illumination. These studies show strong structural transformations as the consequence of UV irradiation, which can only result from local atomic motions. The irradiation obviously generates an effect similar to local heating and to additional stress in the BaTiO_3_ layer under which the material creeps and undergoes downward climbing of dislocations with the in-plane ⟨100⟩ Burger’s vector over the vertical {100} planes.^46^ For this to happen at room temperature, there must be a strong internal stress field driving the motion of defects.

We can hypothesize that a combination of effects take place, involving mainly photostrictive effects and defect driven effects. Photostriction is a light-matter interaction that induces nonthermal dimensional changes in materials.^47,48^ In ferroelectrics, the generally acknowledged mechanism for photostriction is the coupling between the bulk photovoltaic effect and converse piezoelectricity.^47,48^ Additional contributions such as screening of the depolarization field by photogenerated carriers,^18,19^ electronic pressure on atomic bonds,^49^ light-phonon interactions,^27,28^ and weakening of long-range dipolar coupling may also play a role.^50^

Once electron hole pairs are created, carrier separation (and hence the generation of open circuit voltages and of associated electric fields) may arise from internal fields (potential gradients) resulting e.g., from band bending at interfaces and from charge accumulation at domain walls. Another origin for charge separation is the bulk photovoltaic (BPV) effect, present in crystals of noncentrosymmetric lattice, which originates from the asymmetric motion of the nonthermalized photogenerated carriers.^32^ The photovoltaic field arising from the BPV effect in BaTiO_3_ is on the order of 0.1 kV/cm for single crystals^51^ and of 80 kV/cm for 10 nm thick (001) epitaxial films.^52^ The coercive field of our films is of ∼100 kV/cm. Hence, the BPV effect could–in principle—contribute to polarization switching. However, our films contain a mixed *c*- and *a*-domain configuration (with a majority of *c*-domains, as evidenced by the average *c*- and *a*-lattice parameters extracted from XRD) and the pristine state exhibits a random *c*-up/down and *a*-left/right domain distribution (Figure 2b). We therefore expect an overall close-to-zero resulting BPV response as local photovoltages cancel out. Nevertheless, locally, the separation of the photogenerated carriers due to the local asymmetry in each domain generates local electric fields. These local electric fields (local BPV potential) generate local strain/stress and strain/stress gradients (due to converse piezoelectric and flexoelectric couplings, respectively), which add up. Liew et al. reported the occurrence of such local photostrictive effects in a depoled 0.7 Pb­(Mg_1/3_Nb_2/3_)­O_3_–0.3 PbTiO_3_ (PMN–PT) single crystal and a strong macroscopic photostrictive response resulting from their sum.^53^

We propose that the generated local strain/stress fields add up to the local strain/stress fields associated with defects and, together with local thermal heating, are driving forces (stress fields) for the motion of point defects such as oxygen vacancies and of extended defects such as dislocations. We believe that defects play a major role in the effects we observe under UV illumination. Indeed, not all our BaTiO_3_ films on silicon evidence the ferroelastic/ferroelectric switching. Electrochemical potentials (associated with defects) and strain are strongly coupled in mixed ionic-electronic conductors (such as BaTiO_3_ and SrTiO_3_).^54–56^ Strain or stress originating from flexoelectricity has been reported to induce a change in defect concentration (which can be considered as a converse Vegard effect).^54–56^ Overall, the additional local strain/stress fields created by local photostrictive effects as described above, and local stress gradients (converse flexoelectric effect) likely drive local defect motions to minimize electrochemical potentials and eventually lead to the thermodynamically stable phase, bulk BaTiO_3_. Why is the *c*-up orientation of bulk BaTiO_3_ favored? The compressive stress imposed by the SrTiO_3_ template favors a *c*-orientation (which is the main orientation of the nonirradiated film). The asymmetry pre-existing in the nonirradiated film–imprint in the hysteresis loops showing a favored up-polarization and back-switching when poling down (Figure S2)–leads to the final upward orientation.

## Conclusion

We have demonstrated stable irreversible ferroelastic switching from *a*- to *c*-domains and 180° ferroelectric switching (from P_down_ to P_up_ in epitaxial BaTiO_3_ grown on SrTiO_3_-buffered Si substrate by 325 nm-UV laser irradiation. The larger the fluence but also the laser intensity, the more *a*-domains are switched to *c*-domains. The illumination leads to significant structural change, with the relaxation of the film to the bulk *c*-axis BaTiO_3_ structure. We propose that the structural changes are driven by strong strain/stress local fields that lead to point and extended defect motions, eventually leading to a relaxed film. This study highlights the potential of UV lasers as a tool for manipulating ferroelastic and ferroelectric domain in epitaxial BaTiO_3_ thin films on Si substrates for device applications. UV illumination could be used as a postdeposition treatment to heal defects and obtain relaxed films with a vertical monodomain polarization. For this purpose, the defect types (point defect/extended defects, cationic or anionic defects) and defect levels needed to trigger the phase transformation from an inhomogeneous strain state to a uniform relaxed one should be investigated.

## Methods

### Sample Growth

A nominal 20 nm thick BaTiO_3_ layer was epitaxially grown on a 4 nm SrTiO_3_-buffered p^++^ Si substrate by molecular beam epitaxy (MBE) with a DCA R450 equipment. First, an as-received 2 in. p^++^ Si wafer was cleaned with a UV/O_3_ cleaner by applying O_3_ and UV together and immediately loaded in the vacuum chamber. The native SiO_*x*_ layer was removed by vacuum annealing at 870 °C with the help of catalytic Sr. The achievement of a reconstructed 2 × 1 Si surface was monitored by high-energy electron diffraction (RHHED). A SrSi_2_ Zintl phase interface was formed on the reconstructed 2 × 1 Si surface by depositing half of a monolayer of Sr and annealing it at 670 °C under vacuum. The formation of the half monolayer of Sr was confirmed by the 2 × 1 reconstruction pattern observed in RHEED. Then, a nominal 4 nm thick epitaxial SrTiO_3_ layer was grown by codepositing Sr and Ti at 360 °C substrate temperature under a low O_2_ partial pressure of 5 × 10^–8^ Torr. After crystallizing the SrTiO_3_ buffer layer at 490 °C, the BaTiO_3_ layer was grown subsequently by codepositing Ba and Ti at a substrate temperature of 650 °C with an O_2_ partial pressure of 5 × 10^–7^ Torr. Finally, the sample was slowly cooled down (5 K/min) under an O_2_ partial pressure of 5 × 10^–6^ Torr.

### X-ray Characterization

θ–2θ, in plane and phi scans were done using a PanAlytical X’pert Pro diffractometer with Cu Kα_1_ radiation and a one-dimensional detector (PIXcel). Ge (220) monochromator was used to remove the Kα_2_ line. The out-of-plane and in-plane scans were performed at χ angles of 0° and 89.5°, respectively. X-ray reflectivity measurements (XRR) were performed using PanAlytical MPD diffractometer and the simulations of the XRR patterns were carried out with the X’pert reflectivity software.

### Optical Characterization

The optical characterization of the BaTiO_3_ layers was performed by spectroscopic ellipsometry with a J.A. Woollam M2000 system. Measurements were carried out at incidence angles of 60°, 70°, and 75° for a wavelength range of 192–1690 nm corresponding to an energy range of 0.73–6.46 eV. The optical constants *n* and *k* were extracted by fitting the experimental data using a Tauc–Lorentz dispersion model.

### Photolithography

The BaTiO_3_ surface was coated with ∼1.6 μm-thick photoresist (AZ5412E) by spin coating at 4000 rpm for 30 s, followed by prebaking at 90 °C for 3 min. Hollow-square patterns were created by direct laser writing (DLW66 + Heidelberg), exposing the resist to a 375 nm UV laser to define the hollow-shaped pattern. The sample was then developed in AZ726MIF for 1 min, selectively dissolving the exposed resist and leaving the unexposed areas. A 20 nm-thick tungsten layer was subsequently deposited by DC sputtering (Von Ardenne sputter system) onto the patterned resist. Finally, a lift-off process was performed by ultrasonication in acetone for 10 min, removing the photoresist and leaving behind the hollow-square tungsten pattern.

### Surface Morphology Characterization

Scanning electron microscopy imaging was done using a ZEISS Merlin microscope, with an energy of 3 kV and with the in-lens detector. AFM topography images were performed with a Park System NX 10 microscope.

### Raman Spectroscopy

Raman measurements were performed using a Horiba HR-evolution Raman spectrometer equipped with a line grating of 1800 l/mm in the backscattered geometry. The excitation source was a linearly polarized 325 nm continuous wave laser, which was focused through a ×40 objective (Numerical aperture = 0.49), corresponding to a theoretical spot size of ∼810 nm. The incident laser intensity was controlled by an attenuation-adjustable neutral intensity filter. Except otherwise mentioned, the irradiation time used was 500, 250, 150, 60, 30, and 15 s for laser intensity of 0.3, 0.6, 1, 2.5, 5, and 10 mW μm^–2^, respectively. Temperature dependent Raman measurements were performed using a ×20 objective (Numerical aperture = 0.39) in a Microstat Hires environment chamber. The chamber was cooled with liquid nitrogen and a Mercury iTC temperature controller was used to regulate the sample stage temperature.

### Scanning Transmission Electron Microscopy

High-resolution scanning transmission electron microscopy (HR-STEM) was performed using a probe-corrected JEOL ARM 200F microscope operated at 200 kV. The microscope is equipped with a cold field-emission electron gun and a probe aberration corrector, enabling a spatial resolution of 0.8 Å in STEM mode at 200 kV. Cross-sectional lamellae were prepared using a focused ion beam (ThermoFisher Helios Nanolab 600i) following a standard lift-out procedure. The lamellae were oriented along the [110] crystallographic direction of the Si substrate. Imaging was conducted using a high-angle annular dark-field (HAADF) detector, allowing visualization of Ba and Ti atomic columns. To enhance image quality, 10 HAADF images were acquired per data set, using a 21 mrad probe semiangle, a detector range of 90–370 mrad, and a pixel dwell time of 3 μs. The acquired HAADF HR-STEM images were processed using the AbStrain method, as described in ref 43, to quantify strain tensor components relative to the Bravais lattice of BaTiO_3_. This involved correcting each image for scan distortions, sample drift, and pixel calibration errors before alignment and summation, resulting in an enhanced, distortion-free image. Atomic displacements were analyzed using the Relative Displacement approach detailed in ref 43, This method enables the extraction of Ba and Ti substructures and the precise measurement of atomic displacements of Ti atoms with respect to the corresponding adjacent barycenter of the Ba substructure unit cells.

### Piezoresponse Force Microscopy

PFM measurements were performed with a Park Systems NX10 microscope. HQ:NSC 18 (Mikromasch) Pt-coated cantilevers were used. The tips had a typical radius below 30 nm, a resonance frequency of ∼75 kHz and a stiffness of ∼2.8 N/m. A dual-frequency resonance-tracking technique with an external lock-in amplifier (UHFLI, Zurich Instruments) was used to enhance the PFM sensitivity. Images of the vertical and lateral components of the polarization were recorded with drive frequencies of approximately 340 and 650 kHz, respectively. The ac modulation was 0.6 V peak to peak. In all PFM measurements, DC-bias was applied to the PFM tip, and the ac modulation was applied to the bottom electrode.

## Supplementary Material



## Notes

Switching spectroscopy and PFM poling and imaging; lithography process; Power dependent unpolarized Raman spectra; Unpolarized Raman spectra of BaTiO_3_ single crystals; Gaussian fits of Raman modes; Effect of laser fluence on ferroelastic switching; Localized switched regions by UV laser; Electrical reversibility of an irradiated region; Electrical reversibility of a region inside an irradiated region; AFM line profiles of irradiated regions; Temperature dependent Raman spectra; No switching with 405 nm laser; Bandgap measurement (PDF)
