# Supplementary material for: Optically Induced Irreversible Ferroelastic and Ferroelectric Switching in Epitaxial BaTiO3 Films on Silicon
Source: ACS Nano. 2025 Oct 23;19(43):37534–43. doi: 10.1021/acsnano.5c05309 (PMC12593359; doi:10.1021/acsnano.5c05309)
Supplement: Supplementary file 1 [file nn5c05309_si_001.pdf]

Supporting Information

Optically induced irreversible ferroelastic and ferroelectric switching in epitaxial $BaTiO_3$ thin films on silicon

Ibukun Olaniyan[1,2]*, Alfredo Blázquez Martínez[1], Valentin Väinö Hevelke[1,2], Sven Wiesner[1], Rong Wu[1,2], Thanh Luan Phan[1], Robin Cours[3], Nikolay Cherkashin[3], Sylvie Schamm-Chardon[3], Dong-Jik Kim[1], Catherine Dubourdieu[1,2]*

[1]Helmholtz-Zentrum Berlin für Materialien und Energie, Hahn-Meitner Platz 1, 14109 Berlin, Germany

[2]Freie Universität Berlin, Physical and Theoretical Chemistry, Arnimallee 22, 14195 Berlin, Germany

[3]CEMES-CNRS and Université de Toulouse, 29 rue Jeanne Marvig, F-31055 Toulouse, France

Emails : israel.olaniyan@helmholtz-berlin.de; catherine.dubourdieu@helmholtz-berlin.de

Keywords: barium titanate, ferroelastic switching, polarization switching, UV, photoelectric effect, silicon, Raman

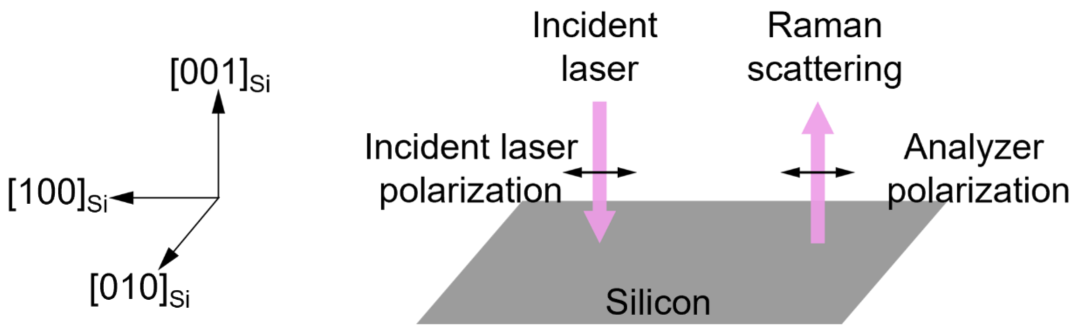

Figure S1: Schematic illustrating the [illegible] Raman scattering geometry.

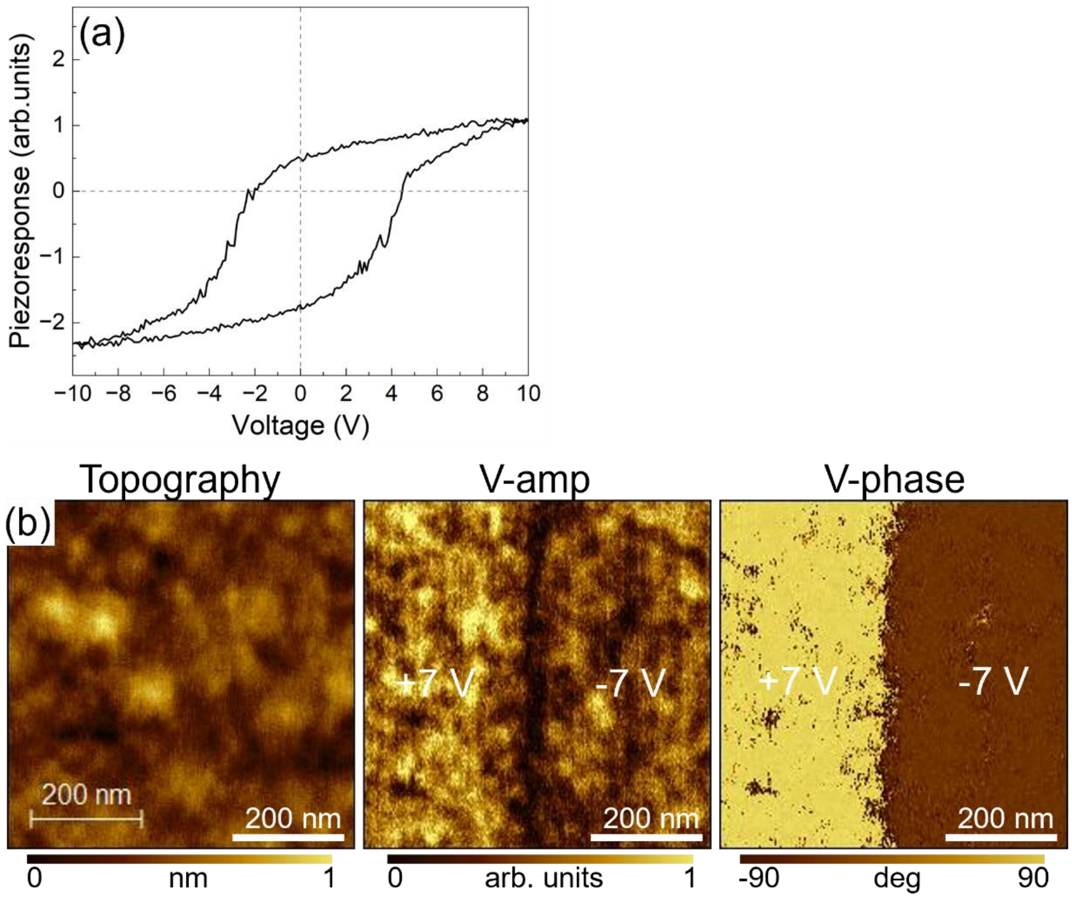

Figure S2: (a) Local PFM hysteresis loop measured on the $BaTiO_3/SrTiO_3/SiO_x/Si$ heterostructure (before irradiation). (b) PFM images showing topography, vertical amplitude (V-amp) and vertical phase (V-phase) of electrically poled regions in the $BaTiO_3/SrTiO_3/SiO_x/Si$ heterostructure.

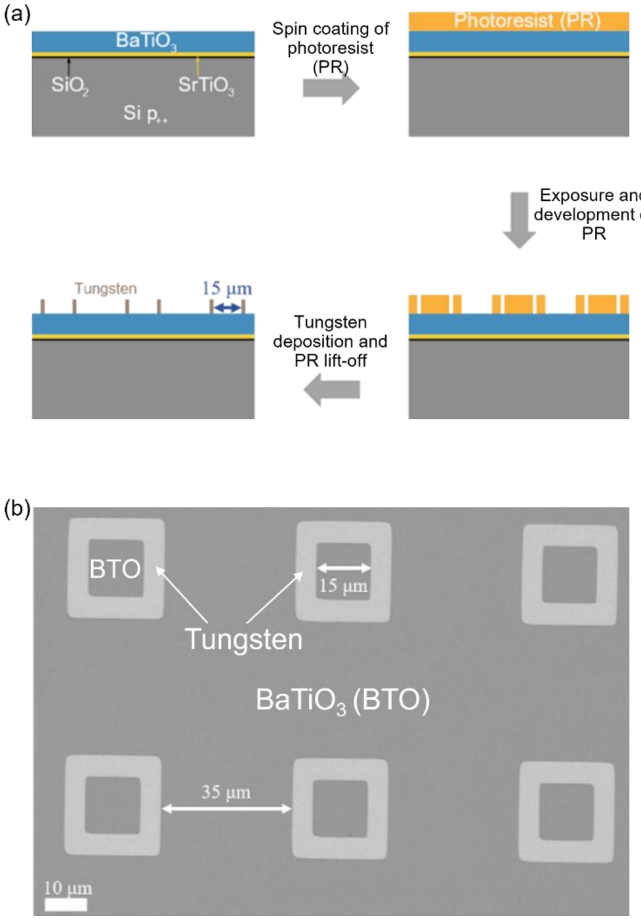

Figure S3: (a) Schematics of the lithography process (b) SEM plan view showing the hollow-square-shaped patterns used to locate the regions of interest on the $BaTiO_3/SrTiO_3/SiO_x/Si$ heterostructure.

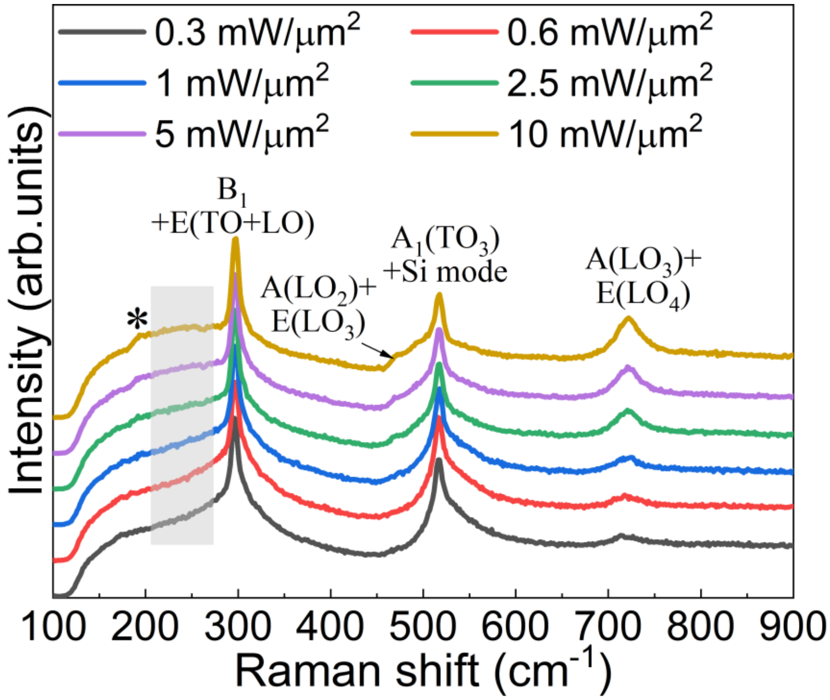

Figure S4: Unpolarized Raman spectra of the $BaTiO_3/SrTiO_3/SiO_x/Si$ heterostructure showing the effect of the UV laser irradiation with different laser intensities at the same fluence of 150 mJ.μm^{-2}.

Noticeable changes in the Raman spectra are seen with an increase in laser intensity: the appearance of a Raman mode at 195 cm^{-1} (indicated with the *) and an increase in the intensity of the $A(LO_2)+E(LO_3)$ and $A(LO_3)+E(LO_4)$ modes. The decrease in the $A_1(TO_3)$ mode is not directly visible here due to its overlapping of the Si mode at ~520 cm^{-1}.

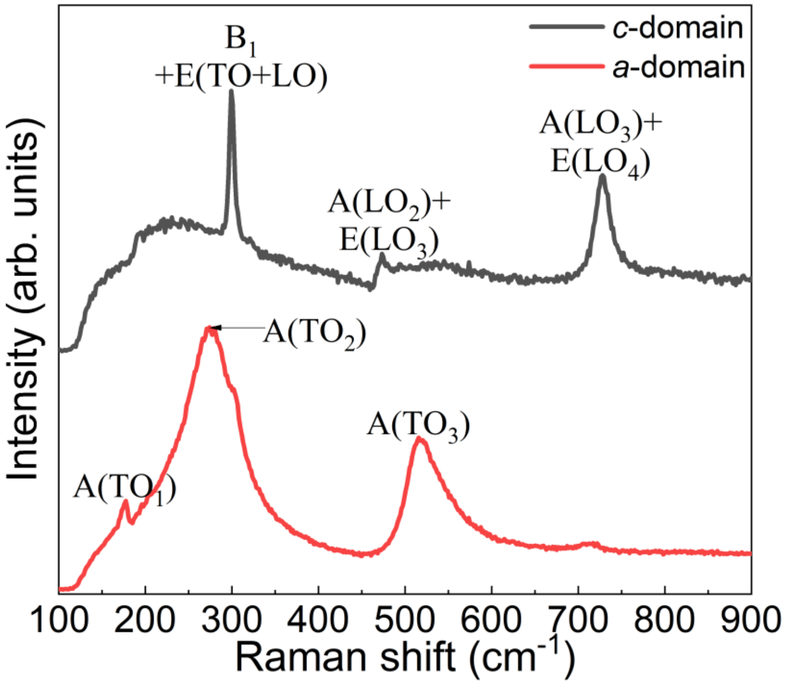

Figure S5: Unpolarized Raman spectra of (001) and (100) $BaTiO_3$ single crystals.

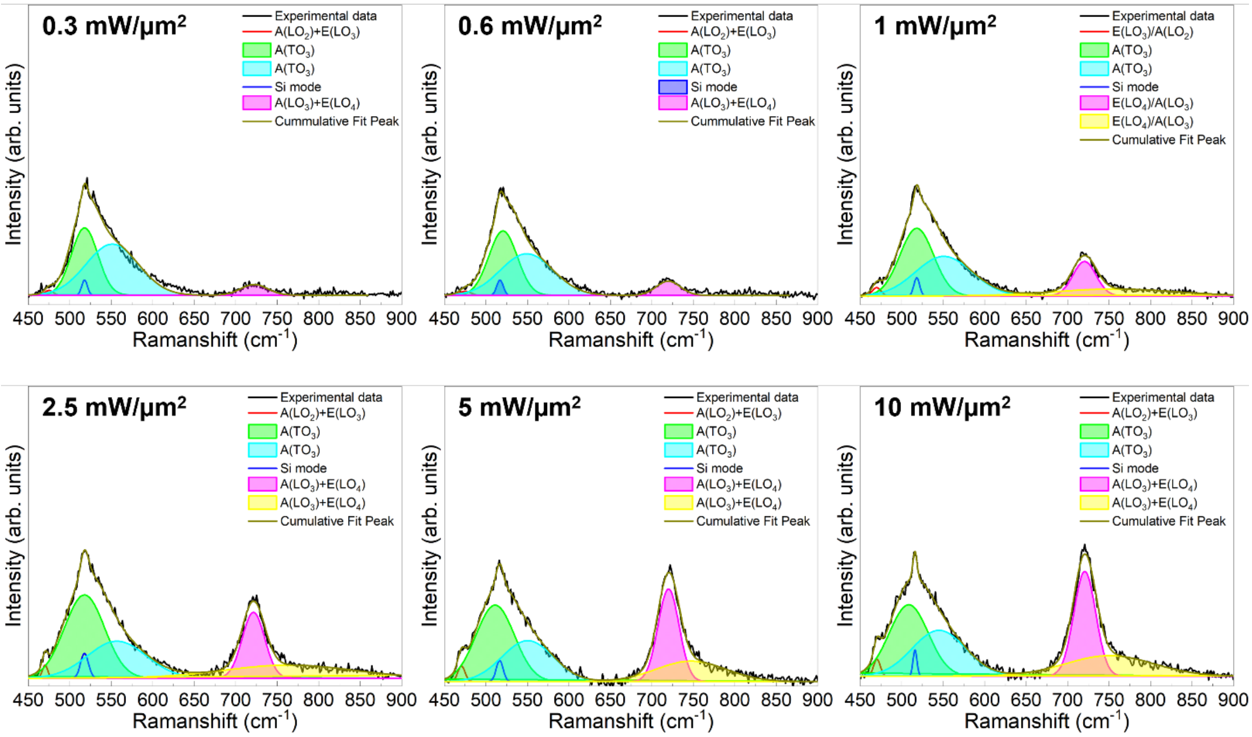

Figure S6: Fitting of the Raman spectra measured at different UV laser power, with a constant fluence of 150 mJ/µm^2.

We show here the fit of the Raman spectra shown in Figure 3a. For each spectrum, a polynomial fit was first applied to subtract the baseline and remove the background contribution. The spectral region between 450 and 900 cm^{-1} was then fitted using Gaussian functions.

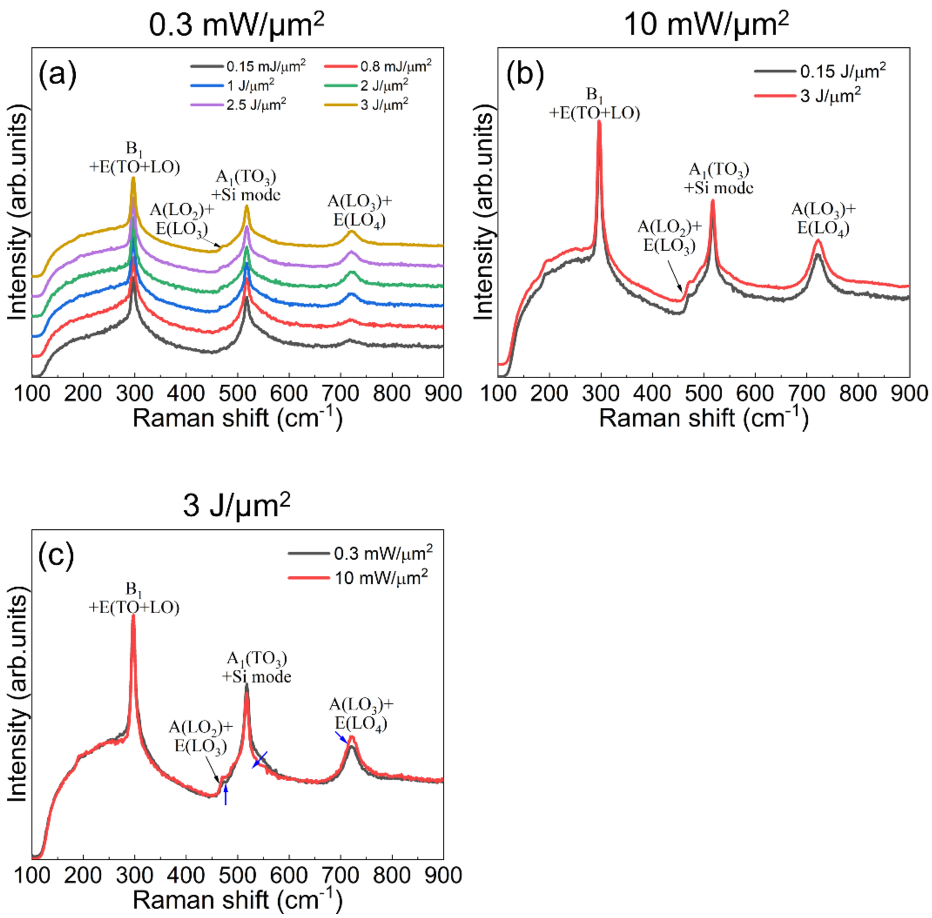

Figure S7: Unpolarized Raman spectra of different regions exposed to UV illumination with different fluences at (a) 0.3 mW/µm^2 and (b) 10 mW/µm^2 laser intensity. (c) Comparison of Raman spectra from regions exposed to a laser fluence of 3 J/µm^2 at different laser intensity. Differences in the spectra are highlighted by blue arrows.

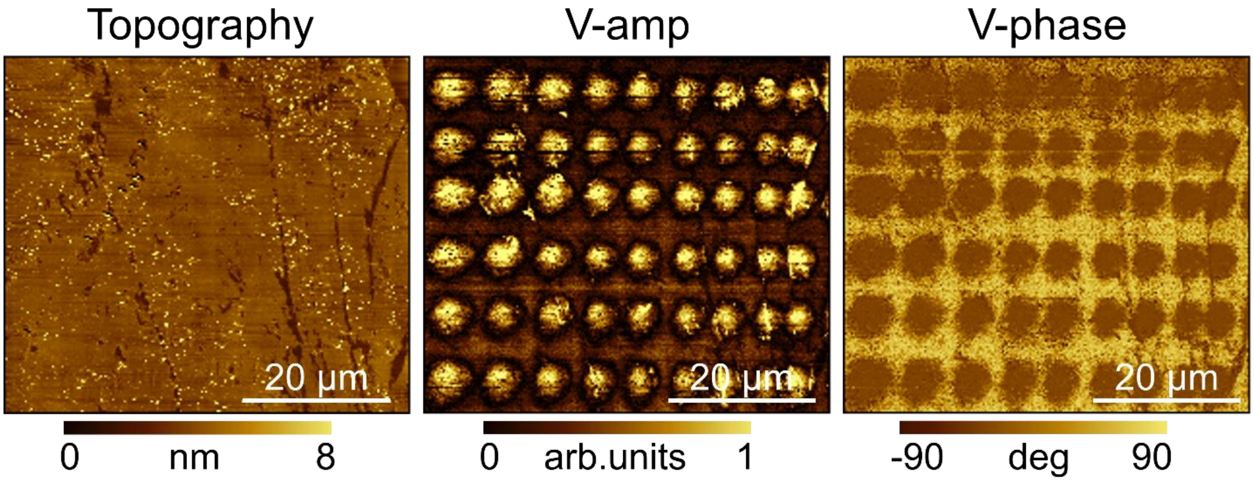

Figure S8: Topography, vertical PFM amplitude (V-amp), and vertical PFM phase (V-phase) images showing an array of localized switched regions induced by UV laser irradiation. The round-shaped regions with enhanced amplitude and dark phase correspond to laser-irradiated areas, created by scanning the sample with a step size of 6 μm under a laser intensity of 10 mW/μm^2.

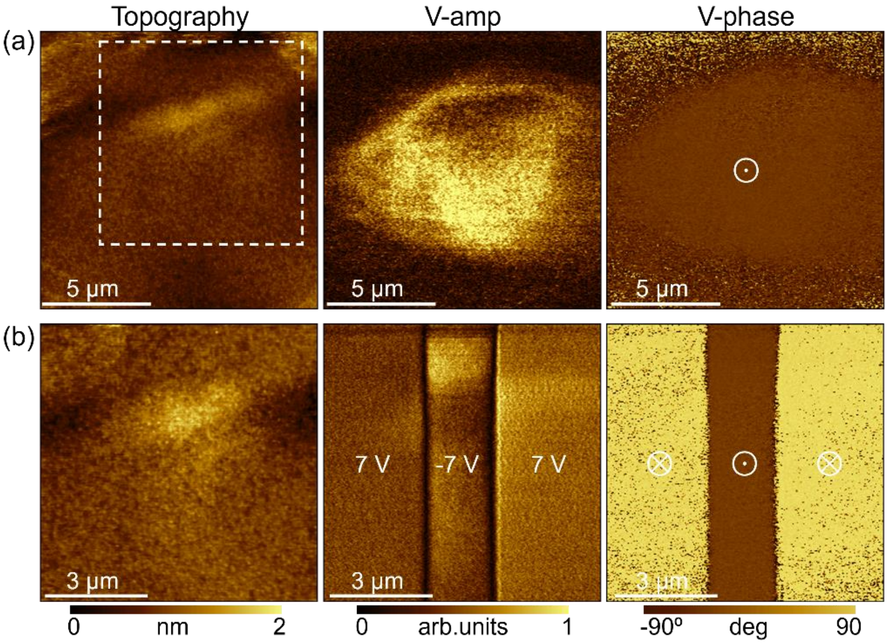

Figure S9: PFM images showing topography, vertical amplitude (V-amp), and vertical phase (V-phase) image of a UV laser irradiated region (a) before and (b) after scanning rectangular areas with an AFM tip bias of +7/-7/+7 V. The rectangular white dashed box indicates where the electric poling was performed.

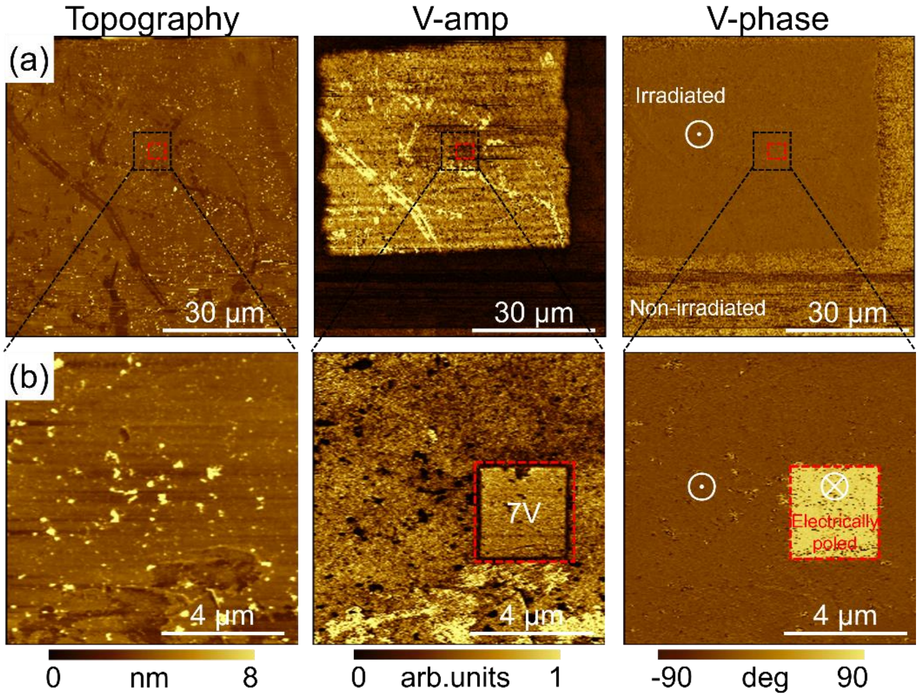

Figure S10. Topography, vertical PFM amplitude (V-amp), and vertical PFM phase (V-phase) images showing (a) both irradiated and non-irradiated regions. The large-area UV laser irradiation was performed using a 2 μm UV-laser step size, and (b) higher magnification image of the irradiated area, including an electrically poled region. The black box in (a) indicates the area shown in (b), and the red box marks the electrically poled region to down orientation using a voltage of 7 V. Back-switching to up-polarization is observed in the red region.

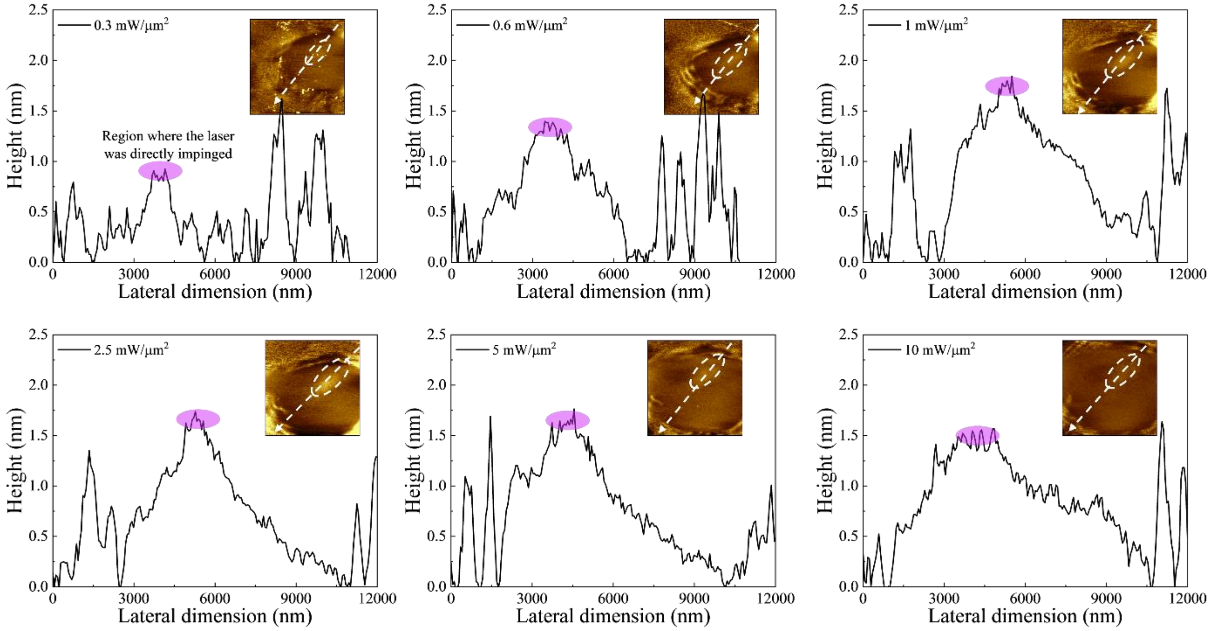

Figure S11: Line profiles of the regions irradiated with varying laser intensities. The purple circles on the line profiles correspond to the center of the irradiation. The insets show the AFM images of each irradiated region. The white dashed circle is the center of the irradiation, and the dashed line is where the line profile is taken.

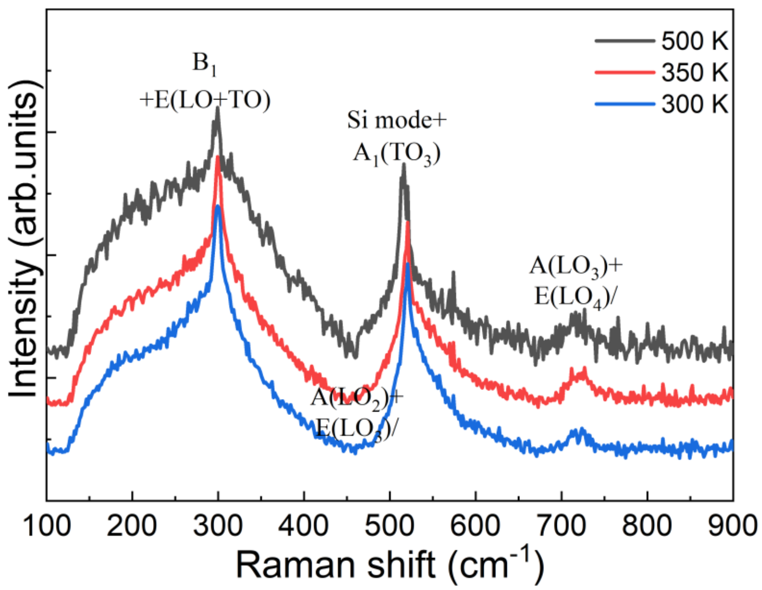

Figure S12: Temperature-dependent Raman spectra of the $BaTiO_3/SrTiO_3/SiO_x/Si$ heterostructure.

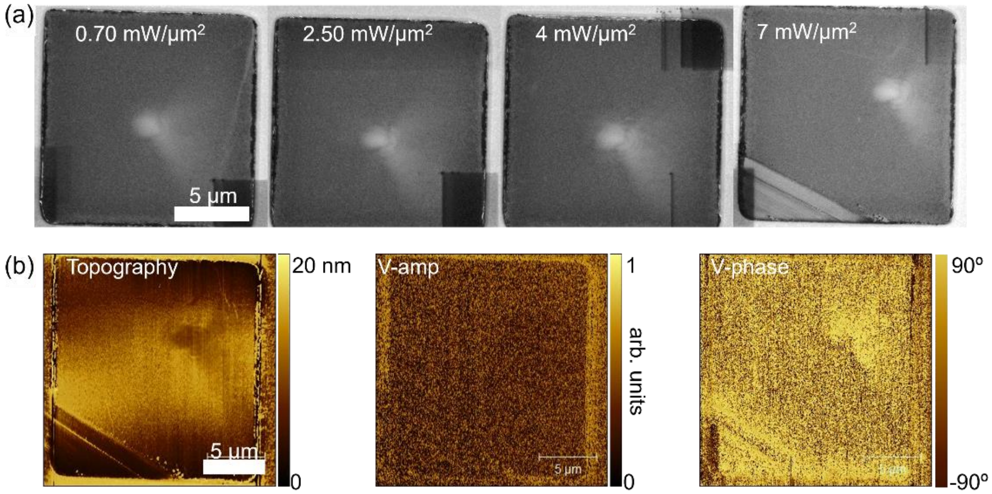

Figure S13: Irradiation of the $BaTiO_3/SrTiO_3/SiO_x/Si$ heterostructure by a 405 nm violet laser. (a) SEM images of the regions irradiated with increasing laser intensities. (b) PFM images showing topography, vertical amplitude (V-amp), and vertical phase (V-phase) of the region irradiated using a laser intensity of 7 mW μm^{-2}.

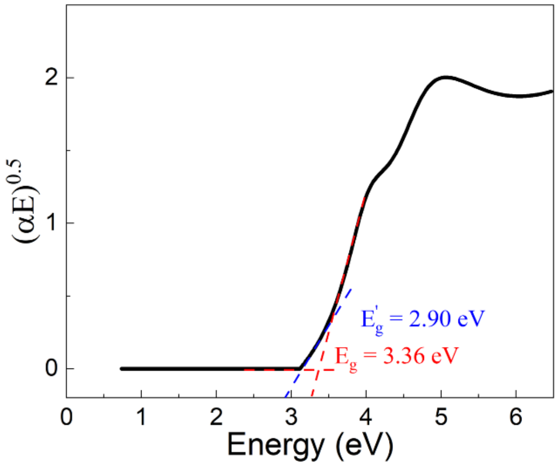

Figure S14: Tauc's plot for the $BaTiO_3$ thin film to estimate the optical bandgap. The extrapolated linear region indicates a bandgap of ~ 3.36 eV and a lower energy of 2.90 eV that likely corresponds to absorption by defects. The broad absorption peak centered around ~ 5 eV is attributed to a higher-energy interband transition from the O 2p valence band to the Ti 3d conduction band.[1]

References

(1) Chernova, E.; Pacherova, O.; Chvostova, D.; Dejneka, A.; Kocourek, T.; Jelinek, M.; Tyunina, M. Strain-Controlled Optical Absorption in Epitaxial Ferroelectric $BaTiO_3$ Films. *Appl. Phys. Lett.* **2015**, *106* (19), 192903.
